# Processing-Induced Changes in Bioactive Compounds and Antioxidant Activity of Orange-Fleshed Sweet Potato (*Ipomoea batatas* L.): Steaming Versus Air-Frying

**DOI:** 10.3390/foods14213637

**Published:** 2025-10-24

**Authors:** Wanida Pan-utai, Naraporn Phomkaivon, Sarn Settachaimongkon

**Affiliations:** 1 Department of Applied Microbiology, Institute of Food Research and Product Development, Kasetsart University, Bangkok 10900, Thailand; 2 Department of Food Chemistry and Physics, Institute of Food Research and Product Development, Kasetsart University, Bangkok 10900, Thailand; ifrnpph@ku.ac.th; 3 Department of Food Technology, Faculty of Science, Chulalongkorn University, Bangkok 10330, Thailand; sarn.s@chula.ac.th

**Keywords:** orange-fleshed sweet potato, thermal processing, carotenoids, phenolic compounds, flavonoids, anthocyanins, antioxidant activity

## Abstract

Orange-fleshed sweet potato (OFSP) is a rich source of carotenoids, phenolics, flavonoids, and starch that are influenced by thermal processing. This study compared the effects of steaming (15–45 min) and air-frying (5–15 min) on the color, bioactive composition, starch content, and antioxidant properties of OFSP peel and flesh to assess processing-induced changes. Unprocessed samples served as the baseline for evaluating percentage and fold changes. Results revealed tissue-specific responses. Steaming preserved flesh brightness (L*, 79) and moderately enhanced carotenoids (0.68 µg/g), anthocyanins (up to 40.5 µg/g in peel), phenolics (2.19–2.27 mg GAE/g), and flavonoids (up to 3.32 mg QE/g). Air-frying induced more pronounced increases in peel bioactives, with carotenoids (4.79 µg/g, 14-fold), phenolics (6.86 mg GAE/g, more than 3-fold), flavonoids (11.75 mg QE/g, more than 20-fold), and transient anthocyanin elevation (61.62 µg/g at 5 min), but prolonged exposure caused degradation in flesh. Starch remained stable in the peel but decreased in the flesh. Antioxidant activity showed similar tissue- and treatment-specific patterns. Steaming preserved structural integrity and bioactive stability, whereas short-duration air-frying maximized peel carotenoids and phenolics. These findings provide insights for optimizing thermal processing to enhance nutritional quality and functional properties in OFSP.

## 1. Introduction

Orange-fleshed sweet potato (OFSP, *Ipomoea batatas* L.) has gained increasing global recognition as a staple crop and functional food due to its rich nutrient profile and potential health-promoting properties [1]. Unlike other sweet potato varieties, OFSP is characterized by its vibrant orange color, reflecting the abundance of provitamin A carotenoids, a critical nutrient for addressing vitamin A deficiency (VAD) in developing regions [2]. This deficiency contributes significantly to blindness, impaired immunity, and increased mortality among children and pregnant women [3]. OFSP also contains high levels of polyphenols, flavonoids, anthocyanins, dietary fiber, and starch, which collectively contribute to its antioxidant capacity and health-promoting attributes [3,4]. These compounds have been associated with anti-inflammatory, hepatoprotective, cardioprotective, and antidiabetic effects, reinforcing the role of OFSP in traditional diets and functional food development [5].

However, the health benefits derived from OFSP are influenced by post-harvest handling and processing. Food processing improves palatability, safety, and shelf life but also alters the concentration, stability, and bioavailability of bioactive compounds [4]. Processing enhances the extractability of certain phytochemicals through the disruption of plant cell walls and conversion of compounds into more bioavailable forms, but it also causes substantial degradation of heat-sensitive molecules such as carotenoids and anthocyanins [6]. Therefore, understanding the effects of processing on the bioactive compounds and antioxidant properties of OFSP is essential for optimizing its nutritional potential.

Thermal processing methods such as boiling, steaming, baking, microwaving, and frying have been extensively studied for their effects on the phytochemical profiles of sweet potatoes. These thermal treatments resulted in significant reductions in β- and α-carotene, with steaming and boiling retaining higher proportions compared to roasting or frying [7]. Steaming significantly enhanced chlorogenic acids and total phenolic content (TPC) in multiple sweet potato varieties, while microwaving yielded the highest DPPH radical scavenging activity [8]. The steaming and microwaving processes improved the antioxidant capacity, whereas boiling caused marked reductions in total polyphenols due to leaching losses [9]. These studies indicated that steaming was superior to boiling for conserving or enhancing bioactive compounds, but results varied depending on sweet potato genotype, flesh color, and process parameters [10].

Air-frying, a novel cooking method that circulates hot air at high speed to mimic deep frying with little or no oil, has emerged as a healthier alternative to conventional frying and gained popularity among consumers seeking reduced fat content while maintaining sensory qualities such as crispness and flavor [11]. From a nutritional perspective, air-frying reduces oil absorption while minimizing the degradation of bioactive compounds compared to deep frying [12]. However, few studies have systematically investigated the impact of air-frying on the contained carotenoids, phenolics, anthocyanins, flavonoids, and antioxidant activity of OFSP, while comparative studies on steaming are also scarce. This knowledge gap is critical given the increasing use of air fryers in household and industrial food preparation.

The scientific literature presents divergent findings and unresolved debates about the optimal processing method for preserving the bioactive profile of sweet potatoes. Some studies suggested that steaming maximizes phenolic retention and antioxidant activity [8,13,14], while others reported that frying or baking yielded comparable or superior outcomes for carotenoid preservation in certain cultivars [6]. Contradictions arise from differences in variety, color, processing duration, and temperature, highlighting the need for a direct, controlled comparison of specific techniques [15]. Purple-fleshed sweet potatoes (PFSPs) have been frequently studied due to their anthocyanin richness, but studies on how modern cooking methods like air-frying influence the phytochemical composition of OFSP are limited.

This study compared the impacts of steaming and air-frying on the physicochemical properties, contained bioactive compounds, and antioxidant activity of OFSP. Changes in color, total carotenoids, anthocyanins, total phenolic content (TPC), total flavonoid content (TFC), starch, and antioxidant capacity were assessed using the DPPH, ABTS, and FRAP assays. This is the first study to directly compare air-frying, a modern, low-oil cooking technique, with steaming, a traditional method recognized for nutrient preservation. The results provide evidence-based insights for dietary recommendations, food industry practices, and consumer choices to globally promote functional foods. Both processing methods significantly influenced the bioactive compounds and antioxidant properties of OFSP, underscoring the need for critical considerations when preparing this nutritionally valuable crop.

## 2. Materials and Methods

### 2.1. Materials

A fresh orange-fleshed sweet potato (OFSP) was purchased from Simummuang Market, Lam Luk Ka, Pathum Thani Province, Thailand. The specimen was identified as *Ipomoea batatas* (L.) Lam, belonging to the Convolvulaceae family, and had orange flesh and pink peel. The sample was preserved at the Sireeruckhachati Nature Learning Park, Mahidol University, Nakhon Pathom Province, Thailand, with assigned voucher specimen number PBM 006446. The orange-fleshed sweet potato tubers utilized in this investigation measured between 11 and 15 cm in length and 4 to 6 cm in diameter, with an average fresh weight ranging from 150 to 200 grams per tuber, thereby ensuring uniformity in size and mass across the experimental sample replicates. The fresh sweet potato was washed with clean water to remove any surface contaminants, followed by air-drying for 3 h. Following the drying process, the sample was separated into the flesh and the peel. Both components were then coarse-milled into uniformly sized particles to ensure consistency for subsequent experimental procedures. The moisture content of OFSP flesh and peel samples was assessed by drying the samples in an oven maintained at 105 °C overnight or until a constant weight was achieved. The results were expressed as a percentage, representing the proportion of water relative to the total sample weight on a wet basis. The moisture contents of the flesh and peel samples were 73.33 ± 0.10% and 78.63 ± 0.45%, respectively, on a wet basis.

### 2.2. Experiments

The OFSP flesh and peel samples were subjected to steaming at 100 °C for 15, 30, and 45 min and air-frying at 160 °C for 5, 10, and 15 min. Fresh, unprocessed samples served as the control (native). Each treatment was rapidly cooled to room temperature, then frozen at −20 °C for 24 h before freeze drying. The dried samples were then milled to achieve a uniform particle size and stored at −20 °C in aluminum foil packages for further analysis. All the experiments were conducted as three biological replicates per treatment.

### 2.3. Analysis

#### 2.3.1. Color

The color of the treated OFSP flesh and peel samples was analyzed using a Datacolor Spectraflash Spectrophotometer (SF 600 plus, Datacolor International, Lawrenceville, NJ, USA). The results were reported in CIELAB coordinates as L* (lightness; 0 = black, 100 = white), a* (chromaticity from green [−] to red [+]), and b* (chromaticity from blue [−] to yellow [+]); all indices are unitless.

#### 2.3.2. Bioactive Compounds

The OFSP samples processed under various treatments were analyzed to determine their carotenoid, anthocyanin, phenolic, and flavonoid contents.

The anthocyanin contents in the flesh and peel samples were determined using a QuEChERS extraction method, as detailed in a previous report with modifications [16]. In summary, 1 g of the sample was homogenized with 10 mL of distilled water and 10 mL of acetonitrile. The mixture was then agitated for three min using a vortex mixer. Subsequently, 1 g of sodium chloride, 1 g of trisodium citrate dihydrate, 0.5 g of disodium hydrogen citrate sesquihydrate, and 4 g of anhydrous magnesium sulfate were added, followed by a one-minute agitation. The resulting supernatant was separated by centrifugation at 3660× *g* for 20 min (Frontier^TM^ 2000 Multi Centrifuges, Ohaus, Parsippany, NJ, USA). The supernatant from the extracts was collected to measure the anthocyanins, with total anthocyanin content determined using a modified pH-differential method [17]. Briefly, a 0.025 M potassium chloride buffer (pH 1.0) or a 0.4 M sodium acetate buffer (pH 4.5) was mixed with the supernatant of the sample. The mixtures were incubated for 10 min at room temperature, and the absorbance was measured at 510 nm and 700 nm. The anthocyanin content was calculated using the following equation, and expressed in micrograms C3G equivalent per gram dry weight (µg C3G/g).

(1) $$Anthocyanin\;content\left(\frac{mg}{L}\right)=\frac{A\times DF\times MW\times 10^{3}\;}{\varepsilon \times \iota }$$

The absorbance, A, was calculated using the equation: A = (A_510_ − A_700_) at pH 1.0 minus (A_510_ − A_700_) at pH 4.5. The dilution factor was denoted as DF. The molecular weight (MW) of cyanidin-3-glucoside was 449.2 g/mol, with 1000 as the conversion from g to mg, and the molar extinction coefficient (ε) was 26,900 L^−1^ M^−1^·cm^−1^. The path length (ι) varied depending on the spectrophotometer type, with values of 0.6 cm for a 96-well microplate and 1 cm for a cuvette spectrophotometer.

The total carotenoid content was determined by extraction with 80% fresh acetone at a ratio of 0.1 g/mL. The supernatant from the extracts was collected to measure carotenoid levels, total phenolic and flavonoid contents, and the antioxidant properties. The total carotenoid content was measured at an absorbance of 450 nm, with the concentration of total carotenoids calculated according to an established method from a previous report [18] with minor modifications.

The total phenolic content (TPC) was determined using the Folin–Ciocalteu assay, a spectrophotometric technique that measures a broad spectrum of phenolic compounds through a colorimetric reaction. Briefly, 20 μL of the sample was mixed with 100 μL of 0.2 N Folin–Ciocalteu solution (SRL, Mumbai, India) and 80 μL of 0.7 M sodium carbonate solution, then incubated at room temperature for 8 min. Next, 50 μL of distilled water was added, and the mixture was incubated at 40 °C for 30 min. The absorbance was measured at 750 nm using a microplate reader (M965+, Microplate Reader, Metertech, Taipei, Taiwan). Gallic acid was used as the standard.

TFC was quantified using the aluminum chloride method, which involves forming a colored complex when the flavonoids react with aluminum chloride, and also measured spectrophotometrically. In brief, 100 µL of the sample was mixed with 100 µL of aluminum chloride solution. The mixture was then incubated at room temperature for 10 min, and the absorbance was measured at 405 nm using a M965+ Microplate Reader (Metertech, Taipei, Taiwan). Quercetin was used as the standard.

The TPC and TFC were expressed as milligrams of gallic acid equivalent per gram of sample (mg GAE/g) and milligrams of quercetin equivalent per gram of sample (mg QE/g), respectively, based on [9] with minor modifications.

#### 2.3.3. Starch

The total starch content was assessed using a Megazyme Kit (K-TSTA-100A, Neogen Corporation, Megazyme, Lansing, MI, USA) in accordance with AOAC Methods 996.11 and 76–13.01 [19]. The starch sample was first gelatinized and then hydrolyzed into maltodextrins through the action of thermostable α-amylase, followed by complete enzymatic conversion to D-glucose utilizing amyloglucosidase. The resulting glucose concentration was quantified colorimetrically via the glucose oxidase/peroxidase (GOPOD) assay at an absorbance of 510 nm. The absorbance values obtained were directly proportional to the starch concentration, facilitating accurate quantification of the total starch content, expressed in grams of starch per 100 grams of sample (g/100 g).

#### 2.3.4. Antioxidant Activity Assessed by the DPPH Assay

The antioxidant capacity was determined using the DPPH radical scavenging assay, with slight modifications to an established protocol [20]. A 100 µL aliquot of the sample extract was combined with 100 µL of a 200 µM DPPH solution (2,2-diphenyl-1-picrylhydrazyl, Sigma-Aldrich, Singapore). The reaction mixture was incubated for 30 min at room temperature in the absence of light to mitigate the risk of radical photodegradation, with absorbance measurements conducted at 517 nm using a microplate reader (M965+, Microplate Reader, Metertech, Taiwan). Ascorbic acid was utilized as the reference standard, with the results expressed in milligrams of ascorbic acid equivalent per gram of sample (mg AAE/g).

#### 2.3.5. Antioxidant Activity Assessed by the ABTS Assay

The ABTS assay was conducted following a modified protocol [21]. The ABTS radical cation was generated by reacting 7 mM of ABTS (SRL, Mumbai, India) with 245 mM of potassium persulfate, and the mixture was incubated in the dark at room temperature for 12 to 16 h. The resulting solution was then diluted to achieve an absorbance of 0.70 ± 0.02 at 750 nm. For the antioxidant assessment, 10 µL of the sample extract was combined with 190 µL of the diluted ABTS solution. After a 5-min incubation in the dark at room temperature, the absorbance was measured at 750 nm using a microplate reader (M965+, Metertech, Taiwan). Ascorbic acid was used as the calibration standard, with the antioxidant capacity expressed as milligrams of ascorbic acid equivalent per gram of sample (mg AAE/g).

#### 2.3.6. Ferric Reducing Antioxidant Power (FRAP) Assay

The antioxidant reducing capacity of the samples was evaluated using the ferric reducing antioxidant power (FRAP) assay with minor modifications [22]. The FRAP reagent was prepared by combining 300 mM acetate buffer (pH 3.6), 10 mM TPTZ (2,4,6-tripyridyl-s-triazine) dissolved in 40 mM HCl, and 20 mM FeCl_3_·6H_2_O in a 10:1:1 (*v*/*v*/*v*) ratio. For the assay, 10 µL of the sample extract was mixed with 190 µL of freshly prepared FRAP reagent and incubated in the dark at room temperature for 30 min. The absorbance was measured at 593 nm using a microplate reader (M965+, Metertech, Taiwan). Ascorbic acid was used as the calibration standard, with antioxidant capacity expressed as milligrams of ascorbic acid equivalent per gram of sample (mg AAE/g).

### 2.4. Statistical Analysis

The processing parameters that induced alterations in the bioactive compounds and antioxidant activity of the OFSP were documented as mean values along with their corresponding standard deviations from three independent biological replicates (*n* = 3), each comprising material from a separate tuber/batch processed, with technical triplicates per assay. Statistical analyses were performed using SPSS software (Version 25.0, SPSS, Inc., Armonk, NY, USA). All the experimental variables were evaluated using Duncan’s multiple range test (DMRT) at a 0.05 significance threshold. The overall chemical profiles of the samples were compared using chemometrics. All the measured parameters were normalized and subjected to multivariate analysis using MetaboAnalyst 6.0 (www.metaboanalyst.ca, accessed on 6 June 2025). Heatmap visualization combined with Pearson’s correlation-based hierarchical cluster analysis (HCA) and partial least squares-discriminant analysis (PLS-DA) was performed to evaluate variations in the sample chemical profile patterns. Chemical parameters with variable importance in projection (VIP) score > 1.0 and *p* ≤ 0.05 were considered as potential discriminative components. Pearson’s correlation analyses among the chemical parameters were performed and depicted by a hierarchically clustered correlation matrix and correlation pattern plots.

## 3. Results

Steaming and air-frying significantly affected the bioactive compounds and antioxidant activity in OFSP. The investigation focused on the changes in color, physicochemical properties, total carotenoids, total anthocyanins, TPC, TFC, starch, and antioxidant properties of OFSPs during processing. A multivariable analysis of all the parameters revealed markers of processing-induced changes in OFSP. Flesh and peel materials were measured for initial moisture contents at 26.68 ± 0.10% and 21.37 ± 0.45%, respectively, on a wet basis. Because moisture strongly influences heat transfer, solute diffusion, and analyte concentration during processing, all compositional results were normalized to a dry-weight (DW) basis.

### 3.1. Effect of Steaming and Air-Frying on Colorimetric Attributes of OFSP

**Figure 1:**
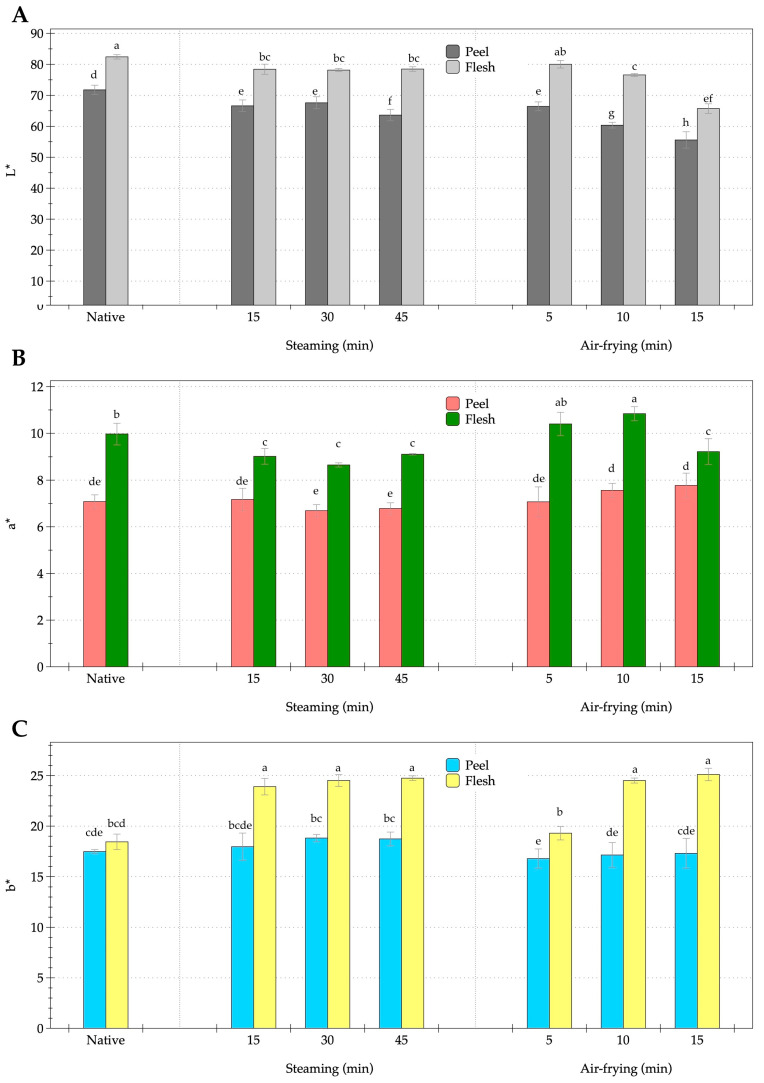
Changes in the color of orange-fleshed sweet potato during steaming and air-frying under various conditions. (**A**), L* (lightness; 0 = black, 100 = white); (**B**), chromaticity coordinates a* (− = green, + = red); (**C**), b* (− = blue, + = yellow). Values represent the mean ± SD (*n* = 3 biological replicates). Different lowercase letters indicate significant differences (*p* < 0.05) among the processing conditions.

Both steaming and air-frying significantly altered the color attributes of the peel and flesh, as measured by color parameters (Figure 1A–C). Steaming produced milder effects on chromaticity compared to air-frying. Figure 1A presents the lightness values (L*). In the native unprocessed samples, the peel and flesh exhibited L* values of 71.76 and 82.41, respectively, indicating that the flesh was inherently brighter than the peel. Steaming for 15 to 45 min preserved flesh brightness, with L* values remaining near 79 and not significantly different from the unprocessed sample. By contrast, the peel showed a progressive decline in lightness, with L* decreasing from 71.76 to 63.61. This reduction in peel lightness resulted from pigment leaching and structural disruption of the epidermal tissues, which increased light absorption. Air-frying resulted in a more pronounced decrease in L*, particularly in the peel, where values dropped to 60.35 at 10 min and 55.58 at 15 min. The flesh also darkened significantly, reaching an L* of 65.71 at 15 min. The redness (a*) values are displayed in Figure 1B. Native flesh exhibited an a* value of 9.97 compared with 7.08 in peel, reflecting its higher carotenoid concentration. Steaming significantly reduced redness in both samples, with flesh decreasing to 9.11 and peel to 6.78 after 45 min (*p* < 0.05). This decline suggested thermal degradation and isomerization of carotenoids, which are particularly sensitive to prolonged moist heat. By contrast, air-frying enhanced redness, particularly in flesh, which reached an a* value of 10.84 after 10 min, surpassing the native level. Both steaming and air-frying significantly increased the yellowness value (b*), as shown in Figure 1C. The unprocessed flesh had a b* value of 18.44, which increased to 24.75 after steaming and 25.11 after air-frying. Peel b* values also increased from 18.73 in both treatments, but remained consistently lower than the flesh. To further assess the impact of processing, tissue color was systematically compared and evaluated, with results summarized in Tables S1 and S2 for the peel and flesh, respectively. In the peel, L* values decreased with increasing processing severity, from 71.76 in the native state to 55.58 after 15 min of air frying (*p* < 0.05). The a* value showed a slight increase after 15 min of air frying, while the b* value was highest following 30–45 min of steaming (18.82–18.73). For the flesh, L* values remained elevated during steaming (approximately 78–82) but declined after 15 min of air frying. The a* value reached its peak after 10 min of air frying, while the b* value increased with prolonged steaming, reaching 24.5–25.1 after 30–45 min. Therefore, steaming preserved lightness while reducing redness, whereas air-frying induced darker coloration but enhanced both redness and yellowness. These findings suggested that thermal processing modulated color attributes differently between the peel and the flesh, with air-frying producing more intense pigmentation changes.

### 3.2. Effect of Steaming and Air-Frying on Bioactive Compounds in OFSP

**Figure 2:**
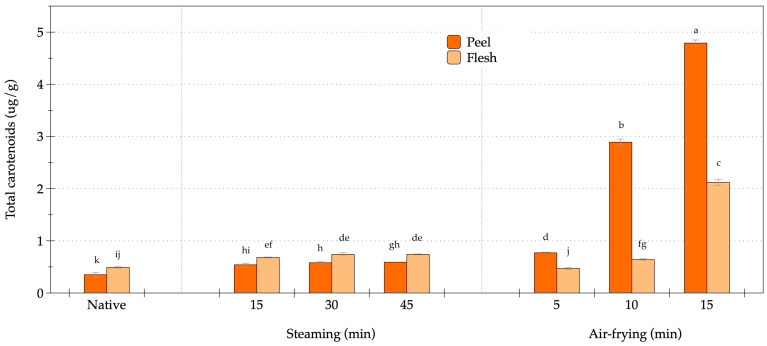
Changes in carotenoid content of orange-fleshed sweet potato during steaming and air-frying under various conditions. Values are expressed as dry weight (DW); data represent mean ± SD (*n* = 3 biological replicates). Different lowercase letters indicate significant differences (*p* < 0.05) among the processing conditions.

The influence of thermal processing on carotenoid accumulation and retention in the peel and flesh of OFSP was investigated (Figure 2). Results revealed significant differences in carotenoid levels between the native, steamed, and air-fried samples. Initial assessments indicated low carotenoid concentrations in native samples, with values of 0.35 µg/g in the peel and 0.49 µg/g in the flesh, classified as the lowest significance category and highlighting the limited extractable carotenoids in the raw material. Steaming resulted in marked enhancements in carotenoid levels compared to the native controls, particularly after 15 min, when concentrations increased to 0.54 µg/g in the peel and 0.68 µg/g in the flesh (*p* < 0.05). These concentrations remained stable at both the 30- and 45-min marks, with the peel registering 0.59 µg/g and the flesh 0.74 µg/g. This stability across varying steaming durations suggested that extended moist-heat exposure did not promote further carotenoid release beyond an initial equilibrium threshold. Conversely, air-frying demonstrated a significant, time-dependent enhancement of carotenoid content. At the 5-min interval, the peel displayed a carotenoid concentration of 0.77 µg/g, while the flesh was measured at 0.47 µg/g, thereby surpassing both the steamed and native control levels. After 10 min, the peel content increased to 2.89 µg/g, peaking at 4.79 µg/g after 15 min as the highest concentration recorded, while the flesh reached 2.12 µg/g (*p* < 0.05). The steamed samples showed higher carotenoid levels in the flesh compared to the peel, but this trend was reversed in the air-fried samples, where the peel accumulated significantly higher carotenoid quantities than the flesh. The peel-to-flesh ratio peaked after 15 min of air-frying, underscoring the dramatic increases in carotenoid extractability associated with this cooking method. Steaming yielded moderate but consistent improvements in carotenoid availability, whereas air-frying facilitated substantial increments that were time-dependent, particularly in the peel tissue. The highest carotenoid concentrations in air-fried peel after 15 min indicated a 14-fold increase compared to the native peel, emphasizing the effectiveness of air-frying in optimizing carotenoid extraction from OFSP.

**Figure 3:**
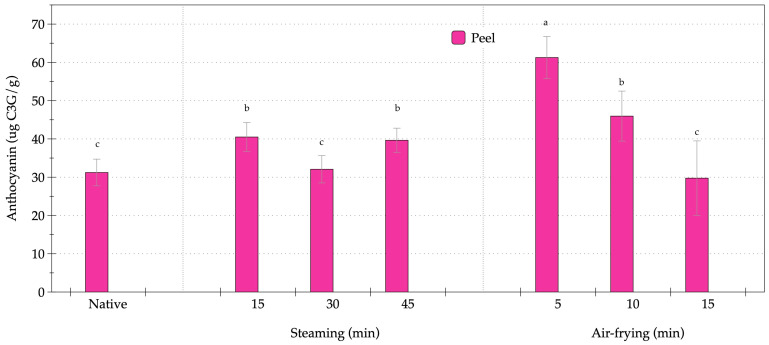
Changes in anthocyanin content of orange-fleshed sweet potato during steaming and air-frying under various conditions. Values are expressed as dry weight (DW); data represent mean ± SD (*n* = 3 biological replicates). Different lowercase letters indicate significant differences (*p* < 0.05) among the processing conditions.

Anthocyanins were elevated in the peel tissue of orange-fleshed sweet potato, with no measurable amounts detected in the flesh (Figure 3). Thermal processing significantly influenced the concentrations of anthocyanins in the OFSP peel. The lowest anthocyanin content was recorded in the native peel at 31.23 µg C3G/g. Steaming enhanced the extractability of anthocyanins compared to the native sample, but the effect was not strictly time dependent. After 15 min, the anthocyanin level increased to 40.50 µg C3G/g (*p* < 0.05), while after 30 min, the level declined to 32.07 µg C3G/g, statistically similar to the raw state. At 45 min, the values increased to 39.64 µg C3G/g, comparable to the 15-min group. These fluctuations suggested that moderate moist heat enhanced anthocyanin release, while prolonged steaming caused partial degradation and structural reorganization, leading to inconsistent results. Air-frying produced a different response. At 5 min, anthocyanins in the peel peaked at 61.62 µg C3G/g, nearly double the level found in the native state, representing the highest value among all the treatments (*p* < 0.05). At 10 min, the concentration dropped to 45.96 µg C3G/g, and at 15 min further decreased to 29.75 µg C3G/g, statistically comparable to the native sample. These findings indicated that anthocyanins were highly sensitive to extended exposure to dry heat, showing an initial surge in extractability during shorter durations, followed by rapid degradation with longer treatments. Steaming promoted moderate and stable increases in peel anthocyanins between 15 and 45 min, whereas air-frying resulted in a sharp initial rise at 5 min, followed by significant losses with extended exposure. Thus, moist heat processing supported retention with less variability, while dry heat provided a transient enhancement and accelerated degradation beyond a certain time threshold.

**Figure 4:**
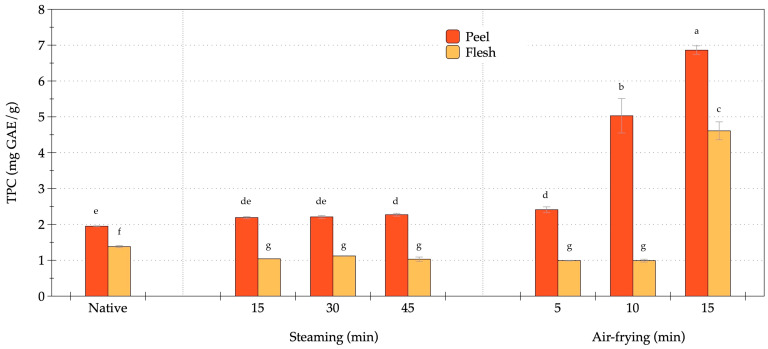
Changes in total phenolic content of orange-fleshed sweet potato during steaming and air-frying under various conditions. Values are expressed as dry weight (DW); data represent mean ± SD (*n* = 3 biological replicates). Different lowercase letters indicate significant differences (*p* < 0.05) among the processing conditions.

The TPC in the peel and flesh of OFSP under steaming and air-frying is presented in Figure 4. In the native sample, TPC was 1.95 mg GAE/g for the peel and 1.38 mg GAE/g for the flesh (*p* < 0.05). The higher concentration of phenolic compounds in the peel compared to the flesh indicated a preferential accumulation in the outer tissues. Steaming resulted in a slight, yet consistent increase in phenolic content in the peel. The values increased from 1.95 mg GAE/g (native) to 2.19 to 2.27 mg GAE/g after steaming at 15 to 45 min, with no significant differences noted among these time points (*p* > 0.05). By contrast, the phenolic content of the flesh remained stable, averaging 1 mg GAE/g, consistently lower than the peel. These results suggested that steaming enhanced the extractability of phenolics in the peel, potentially through mechanisms such as cell wall softening and improved solvent accessibility, while exerting minimal impact on the phenolic profile of the flesh. Air-frying led to an increase in TPC, particularly in the peel tissues. After 5 min, the phenolic concentration in the peel increased to 2.41 mg GAE/g, followed by a significant rise to 5.03 mg GAE/g at 10 min (*p* < 0.05 relative to steaming), and reached a peak of 6.86 mg GAE/g at 15 min (*p* < 0.05), representing more than a 3-fold increase compared to the native peel. The phenolic levels in the flesh remained unchanged at native levels (1.0 mg GAE/g) until 10 min, and then surged to 4.61 mg GAE/g at 15 min (*p* < 0.05). The magnitude of this enhancement indicated that brief air-frying facilitated the liberation of phenolic compounds, while prolonged treatment allowed this effect to penetrate deeper tissues, due to cell disruption and the formation of Maillard-derived phenolic-like compounds. Across all conditions, the peel consistently exhibited a higher phenolic content than the flesh. Steaming promoted a modest and stable retention of phenolics in the peel with negligible effects on the flesh, while air-frying induced a pronounced, time-dependent increase in phenolic content in both tissue types, particularly at the 15-min mark. These findings suggested that phenolic compounds in sweet potato tissues were more effectively augmented by dry-heat-induced structural modifications than by moist-heat processing.

**Figure 5:**
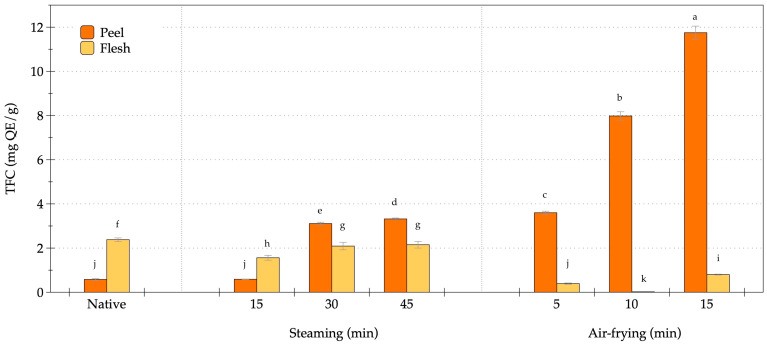
Changes in total flavonoid content of orange-fleshed sweet potato during steaming and air-frying under various conditions. Values are expressed as dry weight (DW); data represent mean ± SD (*n* = 3 biological replicates). Different lowercase letters indicate significant differences (*p* < 0.05) among the processing conditions.

The TFC of OFSP exhibited distinct variations between the peel and flesh in response to thermal processing (Figure 5). The native peel samples presented the lowest baseline value (0.58 mg QE/g), with minimal change after short steaming (15 min; 0.59 mg QE/g). However, prolonged steaming markedly increased TFC, reaching 3.11 and 3.32 mg QE/g after 30 and 45 min, respectively. Air-frying induced a more pronounced elevation, with TFC values of 3.60 and 7.98 mg QE/g at 5 and 10 min, peaking at 11.75 mg QE/g after 15 min, representing a more than 20-fold enhancement relative to the native peel. The flesh exhibited a different trend, with the native state showing higher TFC (2.38 mg QE/g). Steaming induced moderate decreases, particularly at 15 min (1.56 mg QE/g), but values partially recovered with longer steaming durations (2.09–2.15 mg QE/g at 30–45 min). By contrast, air-frying resulted in a dramatic depletion of flesh TFC, declining to 0.39 mg QE/g at 5 min, and further reduced to 0.02 mg QE/g at 10 min, before a rebound at 15 min (0.80 mg QE/g). These findings indicated that air-frying promoted the liberation and accumulation of flavonoids in the peel, and simultaneously accelerated degradation in the flesh, highlighting tissue-specific thermal stability and matrix interactions during processing.

### 3.3. Effect of Steaming and Air-Frying on Starch in OFSP

**Figure 6:**
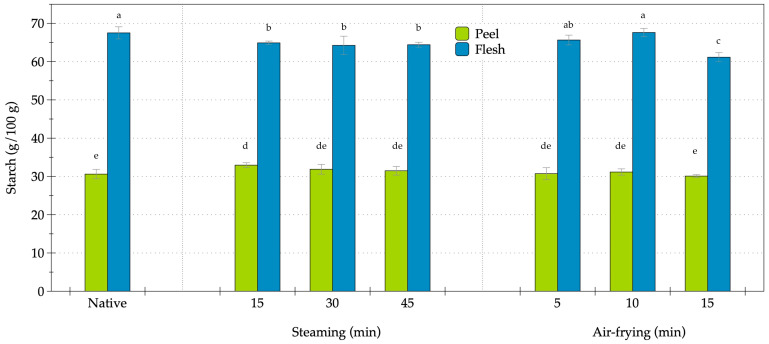
Starch changes in orange-fleshed sweet potato during steaming and air-frying under various conditions. Values are expressed as dry weight (DW); data represent mean ± SD (*n* = 3 biological replicates). Different lowercase letters indicate significant differences (*p* < 0.05) among the processing conditions.

The changes in starch content of OFSP during steaming and air-frying were systematically investigated under various conditions (Figure 6). A substantial difference in starch concentration was observed between the peel and flesh; the peel contained 30.61 g of starch per 100 g, whereas the flesh exhibited a significantly higher concentration of 67.51 g per 100 g. This distinct tissue-specific distribution underscored the role of starch as a dominant component in the internal tissue. Steaming resulted in modest yet statistically significant changes in starch content. In the peel, starch levels showed a slight increase after 15 min (32.95 g/100 g), but subsequently decreased to 31.88 g/100 g and 31.48 g/100 g at 30 and 45 min, respectively. These values were comparable to the native state, indicating a limited impact from the steaming process. By contrast, the flesh demonstrated a consistent decline in starch content, reducing to 64.90 g/100 g, 64.25 g/100 g, and 64.41 g/100 g at 15, 30, and 45 min, respectively, and suggesting the occurrence of partial hydrolysis or leaching, particularly in the starch-rich flesh. Air-frying exhibited a differential effect based on tissue type. In the peel, starch values fluctuated within a narrow range from 30.72 g/100 g at 5 min to 31.15 g/100 g at 10 min, followed by a slight decrease to 30.09 g/100 g at 15 min, indicating the stability of starch in the peel during dry-heat processing. Conversely, the flesh displayed higher variability, with starch content peaking at 67.61 g/100 g after 10 min of air-frying, similar to the native level. However, extended air-frying at 15 min resulted in a significant reduction to 61.15 ± 1.21 g/100 g, as the lowest starch value observed across all the treatments. The starch content in the peel remained stable during steaming and air-frying, exhibiting minor fluctuations around the baseline values. By contrast, the flesh was more sensitive to thermal processing, with consistent reductions occurring during steaming and a marked decline during prolonged air-frying. These findings suggested that the peel showed resilience against processing-induced changes, but the starch in the flesh was more susceptible to hydrolysis and structural modifications.

### 3.4. Effect of Steaming and Air-Frying on Antioxidant Capacities of OFSP

The antioxidant capacities of the peel and flesh during steaming and air-frying treatments are summarized in Table 1. For the peel, the DPPH activity remained stable at 0.16 mg AAE/g across both native and steaming treatments lasting from 15 to 45 min, indicating no significant variations. However, air-frying resulted in a decline in antioxidant activity, decreasing from 0.15 mg AAE/g at 5 min to 0.08 mg AAE/g at 10 min, ultimately reaching a complete loss of activity (0.00 mg AAE/g) by the 15-min mark (*p* < 0.05). By contrast, the flesh consistently exhibited lower DPPH values, maintaining 0.15 mg AAE/g in both native and steamed samples, with a slight decrease to 0.12 mg AAE/g following prolonged air-frying (15 min). Results indicated that DPPH-scavenging phenolic compounds were highly sensitive to heat, especially in the peel tissues.

The peel demonstrated superior ABTS activity compared to the flesh across all treatment conditions. The native peel showed an initial value of 0.38 mg AAE/g, which significantly increased on steaming to 0.53 to 0.60 mg AAE/g (*p* < 0.05). Air-frying resulted in a significant increase in activity, peaking at 1.51 to 1.57 mg AAE/g after 10 to 15 min—a 4-fold enhancement compared to the native sample. On the other hand, the flesh samples displayed lower ABTS values, starting at 0.23 mg AAE/g in native conditions, decreasing during steaming (0.10–0.26 mg AAE/g), and increasing to 1.50 mg AAE/g after 15 min of air-frying (*p* < 0.05). These findings suggested that ABTS-scavenging compounds were released and generated during dry-heat exposure, particularly in the peel tissues.

The ferric reducing antioxidant power (FRAP) assay exhibited the most pronounced heat processing fluctuations. The native peel initially displayed moderate reducing power at 1.05 mg AAE/g, which increased significantly under steaming, reaching values between 7.93 and 8.05 mg AAE/g at 30 to 45 min (*p* < 0.05). By contrast, air-frying led to 10.55 mg AAE/g at 5 min, 31.50 mg AAE/g at 10 min, and peaking at 36.18 mg AAE/g at 15 min (*p* < 0.05), a more than 30-fold increase compared to the native sample. Interestingly, the flesh presented a higher native FRAP activity (3.61 mg AAE/g) than the peel; however, this value diminished slightly during steaming (2.75–3.64 mg AAE/g). During air-frying, the FRAP activity of the flesh experienced a marked decline, from 0.45 to 0.69 mg AAE/g at 5 to 10 min, but partially rebounded to 2.76 mg AAE/g at 15 min.

Antioxidant activity was significantly influenced by tissue type and processing methods. Steaming facilitated moderate enhancements in ABTS and FRAP values for the peel, with minimal effects on the flesh. Conversely, air-frying induced substantial increases in both ABTS and FRAP values for the peel, while concurrently reducing DPPH activity and diminishing the antioxidant response in the flesh, except during prolonged exposure. These observations highlighted peel tissues as primary contributors to antioxidant enhancement under dry-heat conditions, whereas the flesh displayed higher vulnerability to oxidative and structural degradation.

### 3.5. Changes in OFSP Chemical Profiles During Steaming and Air-Frying

These chemometric techniques were employed to determine the changes in chemical profiles occurring during the various processes. Heatmaps were used to visualize standardized abundances (using z-scores), with warmer colors indicating higher relative values. Hierarchical clustering was used to group samples and variables that exhibited similar profiles, thereby revealing patterns driven by the processing conditions. The PLS-DA score plots illustrate the separation of sample groups along latent components, with greater separation suggesting more pronounced multivariate differences. Variables with VIP scores greater than 1 were identified as key factors influencing group discrimination.

**Figure 7:**
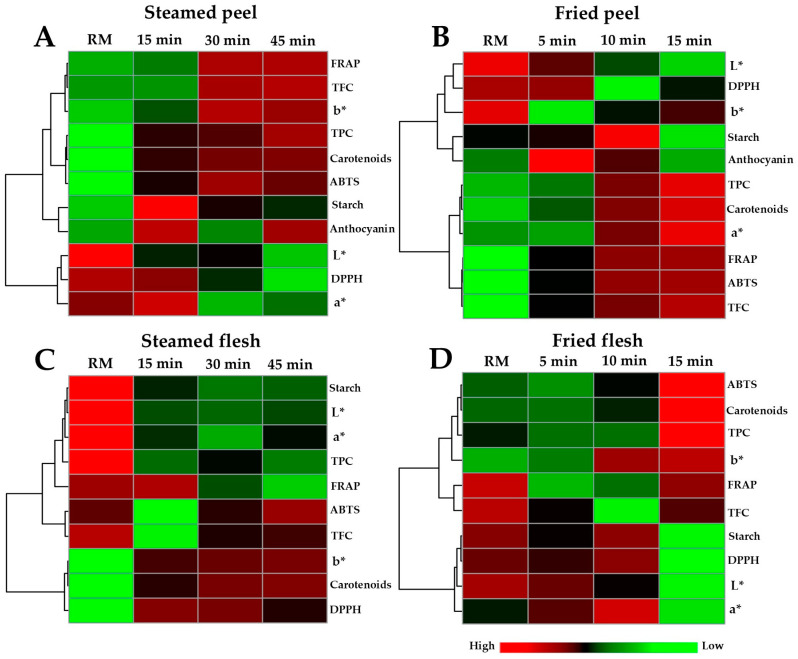
Heatmap visualization and hierarchical clustering of chemical profiles from orange-fleshed sweet potato samples, including steamed peel (**A**), air-fried peel (**B**), steamed flesh (**C**), and air-fried flesh (**D**) collected at various cooking durations. The dendrograms illustrate clustering of the chemical variables based on Pearson’s correlation coefficient with average linkage. Each colored square in the heatmap represents the normalized relative abundances, with red hue indicating higher content and green hue indicating lower content, of the respective chemical parameters.

Pearson’s correlation-based hierarchical clustering combined with heat-map visualization were applied to assess variations in the chemical profiles of OFSP peel and flesh during steaming and air-frying (Figure 7). Decreases in L*, DPPH, and a* values were observed in the OFSP peel subjected to steaming while most other chemical attributes progressively increased over time (Figure 7A). In the flesh samples, steaming led to notable reductions in starch, L*, a*, TPC, and FRAP values, whereas other parameters exhibited a continuous upward trend across the 45-min steaming period (Figure 7C). During air-frying, the OFSP peel samples showed declines in L*, DPPH, and b* values, while TPC, carotenoids, a*, FRAP, ABTS, and TFC values increased steadily throughout the 15-min frying period (Figure 7B). In the flesh parts, frying was characterized by decreases in starch content, DPPH, L*, and a* values, accompanied by marked increases in ABTS, carotenoids, TPC, and b* values as the frying time progressed.

### 3.6. Comparative Chemical Profiling of OFSP Across Different Processing Time Points

**Figure 8:**
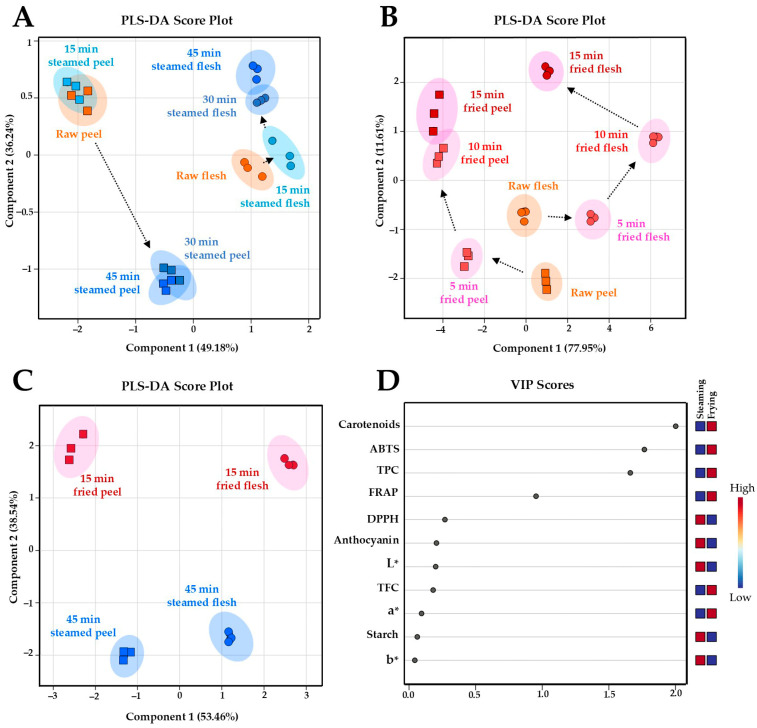
PLS-DA score plots (**A**–**C**) illustrating the effect of thermal processing on orange-fleshed sweet potato chemical profiles. Panels (**A**) and (**B**) demonstrate changes in the chemical profiles of peel and flesh samples across different steaming (15, 30, and 45 min) and air-frying (5, 10, and 15 min) time points, respectively. Dotted arrows indicate the direction of chemical profile shifts with increasing cooking duration. Panel (**C**) demonstrates an overall discrimination of the finished samples obtained from the steaming and frying processes. Important features are organized in descending order of value of variable importance in projection (VIP) scores (**D**). Squares in the VIP score panel express normalized chemical abundance with respect to the color range. The red color indicates a higher content of the corresponding chemical parameter.

Supervised pattern recognitions by PLS-DA were applied to differentiate the chemical profiles among OFSP peel and flesh samples during steaming and frying (Figure 8). To evaluate the influence of steaming, a PLS-DA score plot was constructed using the first two components with a prediction accuracy of 83.33%, *R*^2^ = 0.817, and *Q*^2^ = 0.770 (Figure 8A). Results revealed pronounced alterations in the chemical profile of potato peel samples between 15 and 30 min of steaming, whereas the flesh samples demonstrated only minor yet gradual changes across the entire 45-min steaming period. To assess the impact of air-frying, another PLS-DA score plot was constructed using the first two components with a prediction accuracy of 75.41%, *R*^2^ = 0.832, and *Q*^2^ = 0.767 (Figure 8B). The chemical profile of potato peel samples changed considerably during the first 10 min of frying, with minimal changes thereafter. By contrast, the flesh samples showed continuous and significant alterations over the entire frying duration.

To identify discriminant chemical parameters between the two cooking methods, the final products from each treatment were compared in a separate PLS-DA score plot with a prediction accuracy of 97.85%, *R*^2^ = 0.929, and *Q*^2^ = 0.869 (Figure 8C). The analysis revealed a clear separation among the four groups of cooked potato groups. PLS-DA-derived VIP scores with a value greater than 1.0 and *p* < 0.05 indicated that changes in the content of carotenoids, ABTS radical scavenging activity, TPC, and FRAP (Figure 8D) were the key discriminant parameters differentiating the chemical profiles of cooked OFSP samples resulting from steaming and air-frying, regardless of the tuber part.

### 3.7. Correlation Analysis Among Chemical Parameters

**Figure 9:**
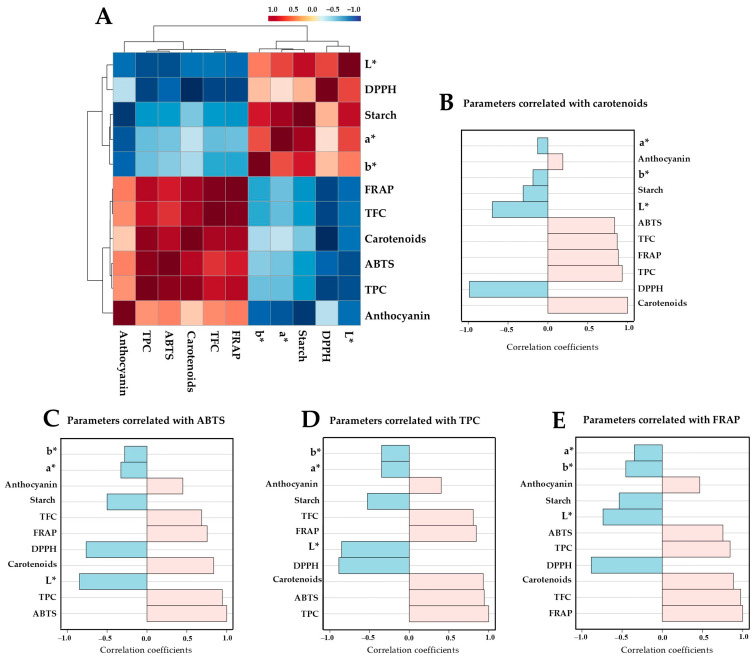
Correlogram of chemical parameters with Pearson’s correlation-based hierarchical clustering (**A**). Correlation coefficients are indicated in each colored cell on the map. The red and blue colors shown in the scale code at the top indicate positive and negative correlations, respectively. Correlation coefficient plots of indicative chemical parameters (**B**–**E**) are represented as horizontal bars, with colors in light pink indicating positive correlations and light blue indicating negative correlations to carotenoids (**B**), ABTS (**C**), TPC (**D**), and FRAP (**E**).

A Pearson’s correlation coefficient matrix among the chemical parameters was constructed and visualized as a correlogram (Figure 9A). The analysis revealed two main clusters comprising positively correlated (red shading) and negatively correlated (blue shading) parameters. Chemical features with PLS-DA-derived VIP scores greater than 1.0, i.e., carotenoid content, ABTS radical scavenging activity, TPC, and FRAP were selected to determine their correlations with the other parameters. A strong association between parameters was considered when the correlation coefficient was greater than 0.5. Results indicated that the carotenoid content in cooked potato samples was positively correlated with TPC, FRAP, TFC, and ABTS values, but negatively correlated with DPPH activity and L* values (Figure 9B). ABTS radical scavenging activity and TPC displayed a similar trend, showing positive correlations with carotenoids, FRAP, and TFC (Figure 9C,D). FRAP was also positively correlated with TFC, carotenoids, TPC, ABTS, and anthocyanin contents (Figure 9E). Notably, ABTS, TPC, and FRAP showed similar negative correlation patterns with DPPH, L*, and starch content of the samples.

## 4. Discussion

This study evaluated the impact of steaming and air-frying on the physicochemical properties, bioactive compound profiles, and antioxidant activity of OFSP. Thermal processing methods influenced the color, total carotenoids, anthocyanins, phenolics, flavonoids, starch content, and antioxidant activity of OFSP, underscoring the importance of processing choices in maximizing nutritional and functional food qualities. Color changes reflected pigment stability and Maillard reactions during thermal processing [23]. Steaming induced minimal reductions in L*, a*, and b* values, consistent with the mild nature of moist-heat processing, which preserved pigments and minimized non-enzymatic browning [24]. By contrast, air-frying resulted in significant color darkening and increased redness (a*), as previously documented for sweet potato and carrot [25]. Higher temperatures accelerated caramelization and Maillard browning, leading to the formation of melanoidins and pigment degradation [26]. Previous studies observed similar color changes, which were linked to thermal processing intensity [23,27]. The color values obtained from the freeze-dried sample powders represent intrinsic pigment composition rather than the visible surface color perceived by consumers. This method enhanced the reproducibility and comparability of pigments, but the color of intact cooked slices may differ due to moisture gradients, surface gloss, and light scattering. Thus, color data should be regarded as indicators of pigment retention rather than direct sensory equivalents. Comparable differences between the powder and surface measurements have been noted in recent studies on food color reflectance and imaging systems [28].

The absolute carotenoid concentrations reported in this study are deliberately conservative relative to investigations that employed saponified, mixed-solvent extraction and HPLC-resolved quantification. Our extraction used polar solvents (acetone and ethanol), which are suitable for extracting more polar carotenoids [29], but the non-saponified protocol applied here can under-recover carotenoids because esterified carotenoids are not hydrolyzed, and pigments embedded within lipid and cell-wall matrices are incompletely liberated. By contrast, saponification followed by hexane partition typically increases apparent recovery by releasing esterified forms and removing interferents; however, these steps can also promote cis–trans isomerization and oxidative degradation if not meticulously controlled [30,31]. Recent analytical overviews reaffirm that HPLC remains the most reliable approach for resolving individual carotenoids and isomers. By contrast, single-wavelength spectrophotometric totals tend to be lower because they integrate fewer species and cannot deconvolute co-absorbing components. Consequently, method choice systematically shifts absolute magnitudes even when relative treatment effects are preserved [32]. For orange-fleshed sweet potato (OFSP), datasets based on exhaustive extraction and HPLC commonly report hundreds of µg/g DW (β-carotene or total carotenoids), with strong cultivar and processing dependence. By contrast, our non-saponified acetone approach yielded lower totals, but ensured comparability across treatments under a single analytical framework. Recent OFSP reports similarly highlight that methodology is a principal source of variance in absolute values [33]. Thermal treatment modulates measurable carotenoids through two opposing mechanisms. At mild–moderate severities, softening of cell walls and disruption of chromoplast membranes facilitate pigment release and improve solvent accessibility, while at higher temperatures or longer times, cis–trans isomerization and oxidative cleavage reduce absorbance and lower recoverable totals. Recent reviews across plant matrices, including roots and tubers, support these dynamics and document process-specific trends for steaming/boiling versus dry-heat modalities [34]. Our results support the pattern observed, OFSP flesh had slight increases or retention at shorter air-frying durations and conservative preservation with steaming, according to this mechanistic balance. Carotenoids, especially β-carotene, are highly sensitive to heat, oxygen, and light [35], and display tissue- and time-dependent behavior. Short-time air-frying maximized extractable carotenoids in the peel, whereas steaming better preserved carotenoids in the flesh across the tested window. Thus, air-frying proved advantageous for enhancing the flavor of the peel, while steaming better preserved the flesh. Steaming better preserves total carotenoids than air-frying, aligning with studies on sweet potato, carrot, pumpkin, and mango [36]. The mechanism centered on the protective effect of steam, which reduced oxidative stress and inhibited isomerization and cleavage of polyene chains [34,37,38]. By contrast, air-frying exposed carotenoids to elevated temperatures and oxygen, likely promoting trans to cis isomerization, epoxidation, and breakdown into volatile compounds [39,40]. The study did not quantify isomer profiles or oxidation products; however, the observed temporal patterns—specifically, peel enrichment during short air-frying and flesh losses with extended exposure—were consistent with a release mechanism initially driven by matrix softening, followed by degradation mediated by heat and oxygen. β-Carotene losses can reach up to 60% under high-temperature dry-heat conditions [41,42]. The matrix effect also plays a role: steaming softens the cell wall, facilitating carotenoid release and bioaccessibility [43]. However, the excessive heat in air-frying counteracted these benefits, resulting in net losses [44].

Anthocyanins are less abundant in OFSP than in purple-fleshed varieties and contribute to antioxidant capacity and visual appeal [6]. Steaming and air-frying induced significant reductions in anthocyanin content, with air-frying causing greater losses [45]. Anthocyanin stability is compromised by high temperature, pH shifts, and oxygen exposure [46]. The mechanisms involve hydrolytic cleavage of glycosidic bonds, oxidative degradation to phenolic acids, and polymerization into brown pigments [47]. Steaming preserves anthocyanins better than boiling or roasting, while the dry environment of air-frying exacerbates their loss [48]. Studies on other anthocyanin-rich crops, such as purple maize and red cabbage, confirmed the sensitivity of these pigments to dry-heat treatment [49]. Phenolic compounds and flavonoids are recognized for their radical scavenging properties and health-promoting effects [47]. Phenolic and flavonoid responses varied depending on tissue type and processing duration, rather than exhibiting a uniform decline. In the peel, air-frying resulted in notable increases in total phenolic content (TPC) and total flavonoid content (TFC) compared to the native state. This increase was consistent with the release of cell-wall-bound phenolics and enhanced solvent accessibility following mild matrix softening. However, prolonged air-frying attenuated or reversed these gains, likely due to progressive oxidation, volatilization, and thermal degradation [47,50,51,52]. Steaming the peel generally maintained or slightly increased extractable TPC and TFC, aligning with the expectation that moist heat facilitates esterified phenolics hydrolysis and limits oxygen exposure [49]. In the flesh, characterized by lower and more soluble phenolics, steaming produced minor changes or slight reductions, while extended air-frying resulted in time-dependent decreases in flavonoids. Mechanistically, the reduction in phenolics and flavonoids was attributed to their thermal instability and susceptibility to oxidation in the presence of air [47,50]. Flavonoid glycosides are particularly sensitive, undergoing deglycosylation and further degradation at high temperatures [51], while the formation of insoluble complexes during Maillard reactions renders some phenolics unextractable by conventional methods [52]. Overall, these findings suggest that processing effects are highly dependent on both tissue type and severity. Early-stage air-frying can enhance phenolics and flavonoids extractability in the peel, steaming better preserves phenolics and flavonoids than dry heat at comparable intensities, and prolonged heating tends to favor degradation and complex formation.

Starch gelatinization and retrogradation govern OFSP texture and nutrition [53,54,55]. Steaming partially gelatinizes starch, increasing water uptake and digestibility [56]. Air-frying, with higher temperatures and lower moisture, promotes more extensive gelatinization and dextrinization, resulting in altered texture and potential increases in glycemic index [57]. Structural studies indicated that air-frying increased resistant starch formation, though the extent depends on temperature and duration [58]. The differences between methods were mechanistically explained by the thermal and moisture gradients imposed during processing, affecting starch granule swelling, leaching of amylose, and enzyme accessibility [57,58]. The flesh and peel of OFSP exhibited initial moisture contents of 73.33 ± 0.10% and 78.63 ± 0.45%, respectively (wet basis). The difference between the two tissues was small, but influenced their response to thermal processing. Steaming retained higher residual moisture, whereas air-frying promoted rapid dehydration and concentration of solids. These variations in water content likely affected starch hydrolysis, matrix softening, and the extractability of heat-labile bioactive compounds, thereby contributing to the observed differences in compositional and antioxidant outcomes between processing methods. Antioxidant activity, measured by the DPPH, ABTS, and FRAP assays, reflected the cumulative effect of carotenoids, phenolics, and flavonoids [59,60]. The increase in FRAP and ABTS in the peel during air-frying, coupled with a simultaneous decrease in DPPH, was consistent with the formation of Maillard-derived reductants and the sensitivities of the respective assays. However, these species were not directly identified, and this is proposed as a plausible explanation rather than a verified mechanism. The differences among the DPPH, ABTS, and FRAP assays reflect their distinct chemistries and media. DPPH is highly sensitive to phenolic structure, steric hindrance, and solvent polarity and can decline when specific radical-scavenging phenolics deteriorate or become less accessible under low-moisture, high-temperature conditions. Recent cross-assay evaluations showed that DPPH often diverges from ABTS/FRAP and correlates less strongly with bulk reducing power [61]. By contrast, the ABTS and FRAP assays quantify a broader pool of reductants and, thus, better capture increases in overall reducing capacity after dry-heat exposure, as summarized in recent methodological reviews [62]. Maillard reaction products formed during dry-heat processing can contribute measurable reducing capacity that elevates ABTS/FRAP even when DPPH-active phenolics decrease, a pattern highlighted in recent Maillard reaction product assessments [63]. The ABTS/FRAP increases alongside DPPH decreases observed here—especially in air-fried peel—were consistent with assay selectivity, Maillard reaction product contributions, and matrix/solvent effects rather than contradictory antioxidant behavior [64]. Our findings indicated that steaming retained higher antioxidant activity than air-frying, consistent with previous research on sweet potato, carrot, and pumpkin [65,66]. The loss of antioxidant activity in the air-fried samples was explained by the degradation of thermolabile phytochemicals [67]. However, some studies observed a paradoxical increase in antioxidant activity after intense heating, attributed to the formation of Maillard reaction products with radical scavenging properties [68]. These neo-formed antioxidants were structurally distinct and did not confer the same health benefits as native compounds [69].

The correlation between retention of carotenoids, phenolics, and flavonoids and total antioxidant capacity has been confirmed in multiple studies. Recent evidence showed strong positive relationships between β-carotene, TPC, TFC, and DPPH/ABTS activities in processed sweet potato, underlining the importance of preserving these compounds through appropriate processing [65]. The differential effects of steaming and air-frying on OFSP nutrients were attributed to several interrelated mechanisms. Steaming functions at lower temperatures and elevated humidity levels, thereby mitigating oxidative stress and thermal degradation [70], while oxygen exposure during air-frying enhances oxidative reactions, thereby expediting the loss of pigments and phenolic compounds [12]. The matrix effect, whereby processing disrupts cell walls, enhances the release of bioactive compounds; however, it also exposes these compounds to potential degradation [66]. The Maillard reaction is more pronounced in air-frying, resulting in the formation of both antioxidant and pro-oxidant compounds [71].

Thermal processing significantly influenced the bioactive compound profile and physicochemical properties of OFSP, with the extent and direction of these changes dependent on both the processing method and the specific compound analyzed. Our results demonstrated that steaming was superior to air-frying in preserving key bioactive compounds, including total carotenoids, anthocyanins, total phenolics (TPC), and total flavonoids (TFC). Steaming retained 85% of total carotenoids, compared to 62% retention in air-fried samples [7]. This differential retention was primarily attributed to the moist heat environment during steaming, which minimized oxidative degradation, prevented isomerization of all-trans-β-carotene, and limited enzymatic activity [72]. Conversely, air-frying subjected OFSP to higher temperatures combined with forced hot air circulation, which promoted oxidative cleavage and isomerization of carotenoids to less bioactive cis forms, reducing both their quantity and bioefficacy [13]. Anthocyanins exhibited a similar pattern, with a 29% reduction following steaming and a 58% reduction after air-frying. These values concurred with previous studies on purple-fleshed sweet potatoes, where steaming preserved 75% of anthocyanins and baking or roasting caused losses exceeding 50% [49]. The intrinsic instability of anthocyanins under high temperatures explained their susceptibility to air-frying, sensitivity to oxygen, and the lack of protective moisture during dry-heat processing [73]. Mechanistically, thermal degradation of anthocyanins involves cleavage of the glycosidic bond, oxidation of the flavylium cation, and subsequent formation of colorless or brown degradation products, which diminish their antioxidant potential and also alter visual quality [47]. The TPC and TFC were also significantly affected by processing. Steaming led to a 12% decrease in TPC and an 8% decrease in TFC, whereas air-frying caused more pronounced losses of 33% and 27%, respectively [74]. A balance between enhanced extractability and degradation explained these observations. Moist heat during steaming disrupts cell walls and matrix structures, facilitating the release of bound phenolic compounds, whereas exposure to high-temperature, dry conditions during air-frying accelerates oxidative degradation and volatilization [34]. This dual effect highlights the importance of controlling both temperature and moisture content to preserve polyphenolic bioactivity in root vegetables.

Thermal processing induced changes in starch properties. Steaming partially gelatinized starch, enhancing water absorption and producing a softer texture, whereas air-frying resulted in more extensive gelatinization and dextrinization [7]. These modifications concurred with previous reports on sweet potatoes and other starchy root crops, where high-temperature, low-moisture conditions favored the breakdown of amylose and amylopectin chains, leading to increased digestibility but reduced structural integrity [74]. Such starch transformations affect the sensory attributes and also influence the glycemic index and postprandial glucose response, linking processing methods to potential nutritional outcomes. The implications of these findings extend beyond basic compositional analysis [64,75,76]. Firstly, this study reinforced the concept that thermal processing is a key determinant of bioactive retention, providing guidance for both industrial and domestic food preparation. Steaming, by preserving bioactive compounds and antioxidant capacity, offers a practical approach for enhancing the functional quality of OFSP-based foods. Secondly, the mechanistic insights into thermal degradation pathways, including carotenoid isomerization, anthocyanin hydrolysis, and phenolic oxidation can assist in targeting strategies for functional food development, such as encapsulation, matrix modification, or controlled drying. Thirdly, changes in starch gelatinization and dextrinization highlight the importance of considering the nutritional and textural properties in designing consumer-friendly, health-promoting products. From a nutritional standpoint, steaming is preferable for preserving endogenous antioxidants and maintaining the textural integrity of the flesh. Conversely, short-duration air-frying enhances the extractability of carotenoids and phenolic compounds in the peel. The peel of orange-fleshed sweet potatoes (OFSP) is often discarded, but its high phenolic content has potential as a functional ingredient—such as in fine flours, snack coatings, or fiber-enriched formulations. The time–temperature–moisture parameters can inform industrial process optimization to balance nutrient retention with sensory quality attributes like color and crispness, while also leveraging peel streams to reduce waste and enhance the nutritional profiles of the products.

## 5. Conclusions

Steaming and air-frying influenced the physicochemical properties, bioactive compounds, and antioxidant activity of OFSP, with tissue-specific responses. Steaming preserved flesh brightness, maintained moderate carotenoid and anthocyanin retention, and stabilized phenolic and flavonoid contents through limited thermal degradation and enhanced extractability from softened cell walls. By contrast, air-frying induced more pronounced color changes, substantially increased carotenoids, phenolics, and flavonoids in the peel, and transiently elevated anthocyanins, reflecting dry-heat-mediated cell disruption and Maillard reaction contributions, though prolonged exposure caused rapid degradation in the flesh. Starch exhibited tissue-dependent stability, with peel unaffected and flesh more susceptible to hydrolysis under extended heat. Antioxidant capacity showed complex patterns. Steaming moderately enhanced ABTS and FRAP in the peel with minimal impact on the flesh, whereas air-frying markedly increased peel antioxidant activity but reduced DPPH and FRAP in the flesh, highlighting the differential thermal sensitivity. Steaming was optimal for preserving intrinsic bioactive content and structural integrity, while short-duration air-frying maximized extractable carotenoids and phenolics, particularly in the peel. These findings provide critical insights for processing strategies to enhance nutritional and functional quality in OFSP, emphasizing the importance of tailoring heat treatments according to tissue-specific stability. Our findings indicated that steaming was the most effective method for preserving the antioxidant capacity and structure of OFSP flesh. Brief air-frying boosted carotenoids and phenolics in the peel, if exposure was limited to prevent flesh degradation. These insights can assist consumers to decide whether to keep the peel and choose appropriate cooking methods, while also guiding the industry to optimize processing conditions and utilize the peels as a nutritious, functional ingredient. However, integrated process–structure–function studies are necessary to enable more precise optimization of time–temperature–moisture regimes for both household and industrial applications to advance the design of nutrient-rich and minimally processed OFSP-based foods.

## Figures and Tables

**Table 1:** Antioxidant properties of orange-fleshed sweet potato during steaming and air-frying under various conditions. Values are expressed as dry weight (DW); data represent mean ± SD (*n* = 3 biological replicates).

Time (min)	DPPH (mg AAE/g)				ABTS (mg AAE/g)				FRAP (mg AAE/g)			
Peel												
Native	0.16	±	0.00	^a^	0.38	±	0.01	^e^	1.05	±	0.10	^fg^
Steaming 15 min	0.16	±	0.00	^a^	0.53	±	0.04	^d^	1.43	±	0.04	^fg^
Steaming 30 min	0.16	±	0.00	^a^	0.60	±	0.06	^c^	7.93	±	0.33	^d^
Steaming 45 min	0.16	±	0.00	^a^	0.56	±	0.01	^cd^	8.05	±	0.20	^d^
Air-frying 5 min	0.15	±	0.00	^b^	0.93	±	0.07	^b^	10.55	±	0.38	^c^
Air-frying 10 min	0.08	±	0.00	^e^	1.51	±	0.10	^a^	31.50	±	1.08	^b^
Air-frying 15 min	0.00	±	0.00	^f^	1.57	±	0.01	^a^	36.18	±	3.94	^a^
Flesh												
Native	0.15	±	0.00	^b^	0.23	±	0.03	^fg^	3.61	±	0.25	^e^
Steaming 15 min	0.15	±	0.00	^b^	0.10	±	0.02	^h^	3.64	±	0.06	^e^
Steaming 30 min	0.15	±	0.00	^b^	0.21	±	0.01	^fg^	3.05	±	0.24	^ef^
Steaming 45 min	0.15	±	0.00	^b^	0.26	±	0.02	^f^	2.75	±	0.11	^ef^
Air-frying 5 min	0.14	±	0.00	^c^	0.18	±	0.02	^g^	0.45	±	0.03	^g^
Air-frying 10 min	0.15	±	0.00	^b^	0.36	±	0.01	^e^	0.69	±	0.01	^g^
Air-frying 15 min	0.12	±	0.00	^c^	1.50	±	0.02	^a^	2.76	±	0.35	^ef^

Different lowercase letters indicate significant differences (*p* < 0.05) among the processing conditions.

## Data Availability

The original contributions presented in this study are included in the article/Supplementary Materials. Further inquiries can be directed to the corresponding author.

## Supplementary Materials

The following supporting information can be downloaded at: https://www.mdpi.com/article/10.3390/foods14213637/s1, Table S1: Changes in peel color of orange-fleshed sweet potato during steaming and air-frying under various conditions; Table S2: Changes in flesh color of orange-fleshed sweet potato during steaming and air-frying under various conditions.

## Abbreviations

The following abbreviations are used in this manuscript:

OFSP	Orange-fleshed sweet potato
TPC	Total phenolic content
TFC	Total flavonoid content
