# Supplementary material for: Processing-Induced Changes in Bioactive Compounds and Antioxidant Activity of Orange-Fleshed Sweet Potato (Ipomoea batatas L.): Steaming Versus Air-Frying
Source: Foods. 2025 Oct 24;14(21):3637. doi: 10.3390/foods14213637 (PMC12608976; doi:10.3390/foods14213637)
Supplement: Supplementary file 1 [file foods-14-03637-s001.zip › foods-3894615-supplementary.pdf]

Processing-Induced Changes in Bioactive Compounds and Antioxidant Activity of Orange-Fleshed Sweet Potato (*Ipomoea batatas* L.): Steaming Versus Air-Frying

Wanida Pan-utai [1,*], Naraporn Phomkaivon [2] and Sarn Settachaimongkon [3]

[1] Department of Applied Microbiology, Institute of Food Research and Product Development, Kasetsart University, Bangkok 10900, Thailand
[2] Department of Food Chemistry and Physics, Institute of Food Research and Product Development, Kasetsart University, Bangkok 10900, Thailand; ifrnpph@ku.ac.th
[3] Department of Food Technology, Faculty of Science, Chulalongkorn University, Bangkok 10330, Thailand; sarn.s@chula.ac.th
* Correspondence: ifrwdp@ku.ac.th

Table S1. Changes in peel color of orange-fleshed sweet potato during steaming and air-frying under various conditions. L* (lightness; 0 = black, 100 = white) and chromaticity coordinates a* (− = green, + = red) and b* (− = blue, + = yellow). Values represent the mean ± SD (n = 3 biological replicates). Different lowercase letters indicate significant differences ($p < 0.05$) among the processing conditions.

Time (min)	L*	a*	b*
Peel			
Native	71.76 ± 1.50 [a]	7.08 ± 0.29 [abc]	17.48 ± 0.19 [ab]
Steaming 15 min	66.60 ± 1.89 [bc]	7.17 ± 0.48 [abc]	17.96 ± 1.35 [ab]
Steaming 30 min	67.58 ± 1.93 [b]	6.69 ± 0.26 [c]	18.82 ± 0.36 [a]
Steaming 45 min	63.61 ± 1.85 [c]	6.78 ± 0.25 [bc]	18.73 ± 0.68 [a]
Air-frying 5 min	66.42 ± 1.44 [bc]	7.07 ± 0.64 [abc]	16.78 ± 0.96 [b]
Air-frying 10 min	60.35 ± 0.93 [d]	7.56 ± 0.30 [ab]	17.14 ± 1.23 [ab]
Air-frying 15 min	55.58 ± 2.65 [e]	7.77 ± 0.53 [a]	17.30 ± 1.48 [ab]

Table S2. Changes in flesh color of orange-fleshed sweet potato during steaming and air-frying under various conditions. L* (lightness; 0 = black, 100 = white) and chromaticity coordinates a* (− = green, + = red) and b* (− = blue, + = yellow). Values represent the mean ± SD (n = 3 biological replicates). Different lowercase letters indicate significant differences ($p < 0.05$) among the processing conditions.

Time (min)	L*	a*	b*
Flesh			
Native	82.41 ± 0.70 [a]	9.97 ± 0.46 [b]	18.44 ± 0.77 [b]
Steaming 15 min	78.42 ± 1.63 [bc]	9.02 ± 0.34 [c]	23.91 ± 0.82 [a]
Steaming 30 min	78.14 ± 0.50 [bc]	8.65 ± 0.09 [c]	24.52 ± 0.59 [a]
Steaming 45 min	78.48 ± 0.73 [bc]	9.11 ± 0.03 [c]	24.75 ± 0.23 [a]
Air-frying 5 min	80.03 ± 1.22 [b]	10.40 ± 0.50 [ab]	19.30 ± 0.67 [b]
Air-frying 10 min	76.58 ± 0.47 [c]	10.84 ± 0.30 [a]	24.51 ± 0.25 [a]
Air-frying 15 min	65.71 ± 1.55 [d]	9.22 ± 0.55 [c]	25.11 ± 0.62 [a]
